# Supplementary material for: Inhibition of SARS-CoV-2 viral entry upon blocking N- and O-glycan elaboration
Source: eLife. 2020 Oct 26;9:e61552. doi: 10.7554/eLife.61552 (PMC7685702; doi:10.7554/eLife.61552)
Supplement: Transparent reporting form [file elife-61552-transrepform.docx]

***eLife’s* transparent reporting form**

We encourage authors to provide detailed information *within their submission* to facilitate the interpretation and replication of experiments. Authors can upload supporting documentation to indicate the use of appropriate reporting guidelines for health-related research (see [EQUATOR Network](http://www.equator-network.org/%20)), life science research (see the [BioSharing Information Resource](https://biosharing.org/)), or the [ARRIVE guidelines](http://www.plosbiology.org/article/info:doi/10.1371/journal.pbio.1000412) for reporting work involving animal research. Where applicable, authors should refer to any relevant reporting standards documents in this form.

If you have any questions, please consult our Journal Policies and/or contact us: [editorial@elifesciences.org](mailto:editorial@elifesciences.org).

**Sample-size estimation**

- You should state whether an appropriate sample size was computed when the study was being designed
- You should state the statistical method of sample size computation and any required assumptions
- If no explicit power analysis was used, you should describe how you decided what sample (replicate) size (number) to use

Please outline where this information can be found within the submission (e.g., sections or figure legends), or explain why this information doesn’t apply to your submission:

All experiments and treatments were carried out at least three times. As this study does not involve patients or animals, power analysis was not performed *a priori*.

**Replicates**

- You should report how often each experiment was performed
- You should include a definition of biological versus technical replication
- The data obtained should be provided and sufficient information should be provided to indicate the number of independent biological and/or technical replicates
- If you encountered any outliers, you should describe how these were handled
- Criteria for exclusion/inclusion of data should be clearly stated
- High-throughput sequence data should be uploaded before submission, with a private link for reviewers provided (these are available from both GEO and ArrayExpress)

Please outline where this information can be found within the submission (e.g., sections or figure legends), or explain why this information doesn’t apply to your submission:

- All error bars represent standard deviation (see individual figures).
- All raw data are explicitly provided in individual plots as discrete data points (see individual figures). Any data normalization procedures are explained in corresponding legends.
- No data has been excluded in individual experiments.
- All data are biological replicates; independent wells with independent cells. However, the virus used in the individual replicates are not always from different lots.
- Critical experiments (including viral preparations) have been independently carried out by more than one of the co-authors of this manuscript.
- Exposure time for all fluorescent micrographs is identical and all images have been treated identically using Zeiss Zen 2012 software. There are no changes to gamma settings and there are no non-linear adjustments in the final images.
- Plasmids developed in our laboratory will be deposited at Addgene (as stated in Methods).

**Statistical reporting**

- Statistical analysis methods should be described and justified
- Raw data should be presented in figures whenever informative to do so (typically when N per group is less than 10)
- For each experiment, you should identify the statistical tests used, exact values of N, definitions of center, methods of multiple test correction, and dispersion and precision measures (e.g., mean, median, SD, SEM, confidence intervals; and, for the major substantive results, a measure of effect size (e.g., Pearson's r, Cohen's d)
- Report exact p-values wherever possible alongside the summary statistics and 95% confidence intervals. These should be reported for all key questions and not only when the p-value is less than 0.05.

Please outline where this information can be found within the submission (e.g., sections or figure legends), or explain why this information doesn’t apply to your submission:

- All error bars represent standard deviation (see individual figures).
- All raw data are explicitly provided in individual plots as discrete data points (see individual figures). Any data normalization procedures are explained in corresponding legends.
- Statistics methods include two-tailed Student’s t-test and one-way ANOVA followed by Tukey post-test.

(For large datasets, or papers with a very large number of statistical tests, you may upload a single table file with tests, Ns, etc., with reference to sections in the manuscript.)

**Group allocation**

- Indicate how samples were allocated into experimental groups (in the case of clinical studies, please specify allocation to treatment method); if randomization was used, please also state if restricted randomization was applied
- Indicate if masking was used during group allocation, data collection and/or data analysis

Please outline where this information can be found within the submission (e.g., sections or figure legends), or explain why this information doesn’t apply to your submission:

This is a basic science study. All treatments were planned in advance of the study and data analysis methods were agreed upon by investigators prior to undertaking individual experiments. All data are presented, with no exclusions.

**Additional data files (“source data”)**

- We encourage you to upload relevant additional data files, such as numerical data that are represented as a graph in a figure, or as a summary table
- Where provided, these should be in the most useful format, and they can be uploaded as “Source data” files linked to a main figure or table
- Include model definition files including the full list of parameters used
- Include code used for data analysis (e.g., R, MatLab)
- Avoid stating that data files are “available upon request”

Please indicate the figures or tables for which source data files have been provided:

All data are presented in individual figures of this manuscript. There are no source data to upload.
